# SARS-CoV-2 productively infects human brain microvascular endothelial cells

**DOI:** 10.1186/s12974-022-02514-x

**Published:** 2022-06-15

**Authors:** Rui-Cheng Yang, Kun Huang, Hui-Peng Zhang, Liang Li, Yu-Fei Zhang, Chen Tan, Huan-Chun Chen, Mei-Lin Jin, Xiang-Ru Wang

**Affiliations:** 1 State Key Laboratory of Agricultural Microbiology, The Cooperative Innovation Center for Sustainable Pig Production, College of Veterinary Medicine, Huazhong Agricultural University, Wuhan, 430070 Hubei China; 2 Key Laboratory of Preventive Veterinary Medicine in Hubei Province, Wuhan, 430070 Hubei China

**Keywords:** SARS-CoV-2, Human brain microvascular endothelial cells, Blood–brain barrier, Inflammatory response, Permeability

## Abstract

**Background:**

The emergence of the novel, pathogenic severe acute respiratory syndrome coronavirus 2 (SARS-CoV-2) has caused a global health emergency. SARS-CoV-2 is highly contagious and has a high mortality rate in severe patients. However, there is very limited information on the effect of SARS-CoV-2 infection on the integrity of the blood–brain barrier (BBB).

**Methods:**

RNA-sequencing profiling was performed to analyze the transcriptomic changes in human brain microvascular endothelial cells (hBMECs) after SARS-CoV-2 infection. Bioinformatic tools were used for differential analysis. Immunofluorescence, real-time quantitative PCR, and Western blotting analysis were used to explore biological phenotypes.

**Results:**

A total of 927 differentially expressed genes were identified, 610 of which were significantly upregulated while the remaining 317 were downregulated. We verified the significant induction of cytokines, chemokines, and adhesion molecules in hBMECs by SARS-CoV-2, suggesting an activation of the vascular endothelium in brain. Moreover, we demonstrated that SARS-CoV-2 infection could increase the BBB permeability, by downregulating as well as remodeling the intercellular tight junction proteins.

**Conclusions:**

Our findings demonstrated that SARS-CoV-2 infection can cause BBB dysfunction, providing novel insights into the understanding of SARS-CoV-2 neuropathogenesis. Moreover, this finding shall constitute a new approach for future prevention and treatment of SARS-CoV-2-induced CNS infection.

**Supplementary Information:**

The online version contains supplementary material available at 10.1186/s12974-022-02514-x.

## Background

Coronaviruses are a group of enveloped, positive-sense, single-stranded RNA viruses that primarily target the human respiratory system [1]. They also have neuro-invasive capabilities, and can spread from the respiratory tract to the central nervous system (CNS) [2]. The coronavirus disease 19 (COVID-19) pandemic is caused by the severe acute respiratory syndrome coronavirus 2 (SARS-CoV-2) [3]. Multiple studies suggest that febrile seizures, anosmia, ageusia, ataxia, and encephalitis may be early signs and manifestations of COVID-19, indicating that this virus may also have neurotropic and neuro-invasive capabilities [4, 5]. However, the mechanisms by which SARS-CoV-2 enters the host brain and causes neuroinflammatory responses are poorly understood. Identification of the specific host molecules involved in these essential steps is urgently needed to clarify these mechanisms.

The blood–brain barrier (BBB), formed by brain microvascular endothelial cells (BMECs), astrocytes, microglial cells, pericytes, and microvasculature, is the most important physiological barrier in mammals [6]. As the interface between the bloodstream and brain, it is essential for restricting the entry of many circulating pathogens, toxins, compounds, inflammatory factors, and immune cells into the CNS, thereby maintaining brain homeostasis [7]. BMECs are the major components of the BBB, and are characterized by the expression of tight junction (TJ) proteins, including claudins (CLDNs), occludin (OCLN), and cytoplasmic zonula-OCLN family members (such as TJP1, TJP2, and TJP3) [8]. Reducing TJ proteins expression or changing their distribution can increase the BBB permeability, which is an important indicator of the BBB dysfunction [9]. Cell adhesion molecules, which are cell surface glycoproteins that facilitate cell–cell interactions, cell adhesion, and migration, are widely distributed in a variety of endothelial cells, including BMECs [10]. Vascular endothelial cell adhesion molecule 1 (VCAM-1), intercellular cell adhesion molecule 1 (ICAM-1), and CD44 are widely believed to promote the migration of leukocytes or monocytes to the infection site [11].

The evidence that BMECs were infected by SARS-CoV-2 was obtained in the brains of SARS-CoV-2-infected patients, as well as in mouse and hamster models. Recent studies have revealed that the main protease of SARS-CoV-2 (Mpro) cleaves NF-κB essential modulator (NEMO). By ablating NEMO, Mpro induces the death of hBMECs and the occurrence of string vessels in mice [12]. And it was also reported that SARS-CoV-2 spike protein caused BBB dysfunction by inducing degradation of endothelial TJ proteins [13, 14]. However, the hBMECs responses to this process are poorly understood. Here, we applied RNA-seq and bioinformatic approaches to identify potential host mRNAs which are activated in hBMECs after SARS-CoV-2 infection. This is the first study to show that SARS-CoV-2 exhibits the ability to penetrate and disrupt the BBB in the hBMECs in vitro model, which shall extend the current knowledge regarding the SARS-CoV-2 neuropathogenic, as well as provide new clues for better prevention and therapies to this disease.

## Methods

### Cell lines and viruses

The hBMECs were kindly provided by Prof. Kwang Sik Kim in Johns Hopkins University School of Medicine, and this cell line is commonly used in the in vitro model of blood–brain barrier, especially in the field of meningitis [15–18]. Immortalized hBMECs were cultured in RPMI1640 medium supplemented with 10% fetal bovine serum (FBS), 2 mM l-glutamine, 1 mM sodium pyruvate, essential amino acids, non-essential amino acids, vitamins, penicillin, and streptomycin (100 U/mL) [19, 20], and then incubated at 37 °C in a humidified atmosphere containing 5% CO_2_.

SARS-CoV-2 strain Wuhan-Hu-1 (NC_045512) was obtained from the Wuhan Institute of Virology, Chinese Academy of Sciences. Strain WBP-1 (EPI_ISL_1615558) was developed in this study. *Chlorocebus sabaeus* (Green monkey) VeroE6 (female, RRID: CVCL_YQ49) were purchased from American Type Culture Collection (ID: ATCC CRL-1586). VeroE6 cells were cultured in Dulbecco’s modified Eagle medium (DMEM) supplemented with 10% FBS at 37 °C in a humidified CO_2_ incubator. The SARS-CoV-2 virus stocks were prepared on Vero cells and 50% tissue culture infective doses (TCID_50_) were calculated using the Reed–Muench formula. All experiments involving live viruses were performed in a biosafety level 3 (BSL3) facility in the Huazhong Agricultural University.

### SARS-CoV-2 infection of hBMECs

Confluent primary hBMEC monolayer was grown in 12-well plates, then washed three times with serum-free 1640 medium before infection at a multiplicity of infection (MOI) of 1. After 1 h of virus adsorption at 37 °C and 5% CO_2_, the cultures were washed twice with serum-free 1640 medium to remove unbound virus, then cells were cultured in 2% fetal bovine serum 1640 medium at 37 °C with 5% CO_2_ for 24 h and 72 h. Finally, cells were washed three times with pre-chilled phosphate-buffered saline (PBS), and subjected to RNA extraction using either a TRIzol reagent (Invitrogen, Carlsbad, CA, USA) or RIPA (Epizyme, Shanghai, China) buffer with a protease inhibitor cocktail (GlpBio, Montclair, CA, USA) for Western blot analysis.

### SARS-CoV-2 infection of mice

For the animal experiments, specific pathogen-free, 12-month-old, female Balb/c mice were obtained from Laboratory Animal Services Centre, Huazhong Agricultural University. All experiments were performed at the BSL-3 core facility at the Huazhong Agricultural University. After intraperitoneal injection with tribromoethanol (Avertin; 250 mg/kg), each mouse was intranasally inoculated with SARS-CoV-2 stock virus at a dose of 10^5^ TCID_50_ in 50 μL DMEM. At 5 d post-inoculation, the mice were euthanized, and their brains were collected [21].

### RNA-sequencing and bioinformatic analysis

Total RNA extraction was performed using the TRIzol reagent, following manufacturer’s instructions. RNA quantity and quality were assessed using a NanoDrop 2000 spectrophotometer (NanoDrop Technologies, Wilmington, DE, USA) and a Bioanalyzer 2100 system (Agilent Technologies, CA, USA). RNA contamination was assessed by 1.5% agarose gel electrophoresis.

For the library preparation, 1 μg of RNA per sample was used. The mRNA was obtained from the total RNA by using poly(T) oligo-attached magnetic beads. Sequencing libraries were generated from the purified mRNA by using the VAHTS Universal V6 RNA-seq Library Kit for MGI (Vazyme, Nanjing, China), following the manufacturer's recommendations, with unique index codes. Library quantification was assessed using a Qubit 3.0 Fluorometer (Life Technologies, Carlsbad, CA, USA) and Bioanalyzer 2100 system (Agilent Technologies, CA, USA). Subsequently, sequencing was performed on the MGI-SEQ 2000 platform by Frasergen Bioinformatics Co., Ltd. (Wuhan, China). The transcriptomic data were deposited to the BioProject database of the National Center for Biotechnology Information (NCBI; accession number PRJNA663975).

Low-quality reads, such as reads with adaptor sequences, reads with > 5% N, or bases with quality < Q20 (percentage of sequences with sequencing error rates < 1%), were removed using Perl script. The clean reads were mapped to the human genome (http://ftp.ncbi.nlm.nih.gov/genomes/all/GCF/000/001/405/GCF_000001405.39_GRCh38.p13) by using HISAT2 [22]. New transcripts were predicted from the genome alignment by using StringTie [23], then reads were mapped to the merged transcriptome set by using bowtie2 [24]. The normalized expression level (FPKM) of each gene and transcript was quantified using RSEM [25]. Genes that were differentially expressed between sample groups were identified using DESeq2 [26]. The false discovery rate (FDR) was used to identify the threshold of the *P*-value in multiple tests. Here, only genes with log_2_ (fold change)| ≥ 1 and FDR significance score (*p*_adj_) < 0.01 were used for subsequent analyses.

Differentially expressed genes were compared against various databases for functional annotation. We compared the plant-specific sequences from the NCBI-nr database and the Swiss-Prot database by using basic local alignment search tool (BLAST) x with an e-value cut-off of 10^–5^. The best BLAST hit based on the bit score was used for subsequent functional annotation. Gene Ontology (GO) annotation was performed based on the correspondence between the genes in the NCBI GO annotations. The database of this correspondence was obtained from https://ftp.ncbi.nlm.nih.gov/gene/DATA/gene2go.gz. Kyoto Encyclopedia of Genes and Genomes (KEGG) pathway annotation was performed using BLAST x against plant-specific sequences from the KEGG database. GO and KEGG enrichment analyses were performed using the hypergeometric test as implemented in the R phyper function.

### Reverse transcription and real-time quantitative polymerase chain reaction (RT-qPCR)

Reverse transcription PCR was performed with 1 μg aliquots of total RNA from each sample and was followed by complementary DNA (cDNA) synthesis using the HiScript II Q Select RT SuperMix for qPCR (+gDNA wiper) (Vazyme, Nanjing, China). RT-qPCR was performed with a QuantStudio 3 RT-qPCR System (Applied BioSystems, Foster City, CA, USA) using 2X M5 HiPer SYBR Premix EsTaq (Mei5 Biotechnology, Beijing, China), according to the manufacturer’s instructions and using the primers listed in Additional file 1: Table S1. The expression levels of the target genes were normalized to those of RPL13A. Each assay was performed three times independently.

For SARS-CoV-2 RT-qPCR, 100 ng of RNA was used as a template for the amplification of selected genes by using TransScript® II Probe One-Step RT-qPCR SuperMix (TransGen Biotech, Beijing, China). Average values from duplicates of each gene were used to calculate the viral genomic copies. Sequences of the primers targeting the SARS-CoV-2 RdRP gene were as follows: 5′-CAATGGTTTAACAGGCACAGG-3′ (forward) and 5′-CTCAAGTGTCTGTGGATCACG-3′ (reverse). RT-qPCR was performed following the manufacturers’ instructions.

### Western blot analysis

hBMECs were collected and lysed in RIPA buffer with a protease inhibitor cocktail, and centrifuged at 12,000 rpm for 10 min at 4 °C to remove insoluble cell debris. The concentration of the soluble protein in the supernatant was measured using the bicinchoninic acid protein assay kit (New Cell & Molecular Biotech, China) and used for subsequent Western blot analysis. Aliquots of each sample were separated by 12% sodium dodecyl sulfate polyacrylamide gel electrophoresis, and then transferred to polyvinylidene difluoride membranes. The blots were blocked with 5% bovine serum albumin in Tris-buffered saline containing Tween-20 at room temperature for 1 h, and then incubated overnight at 4 °C with primary antibodies against TJP1 (Abcam, ab216880, 1:1000, Cambridge, MA, USA), OCLN (Abcam, ab31721, 1:1000, Cambridge, MA, USA), CLDN5 (Abcam, ab131259, 1:1000, Cambridge, MA, USA), VCAM1 (Abcam, ab174279, 1:1000, Cambridge, MA, USA), ICAM1 (Proteintech, 60299-1-Ig, 1:1000, Chicago, IL, USA), CD44 (Proteintech, 15675-1-AP, 1:1000, Chicago, IL, USA), and β-actin (Proteintech, 66009-1-Ig, 1:5000, Chicago, IL, USA). The blots were subsequently washed and incubated with horseradish peroxidase-conjugated anti-rabbit (Biodragon, BF03001, 1:5000, Beijing, China) or anti-mouse IgG (Biodragon, BF03008, 1:5000, Beijing, China) at 37 °C for 1 h and visualized using an electrochemiluminescence reagents (Bio-Rad, Hercules, CA, USA). Densitometry analysis was analyzed in three independent blots by using Image Lab software version 5.2.1 (Bio-Rad, Hercules, CA, USA).

### Immunofluorescence assay

hBMECs grown in 6-well dishes were fixed with 4% paraformaldehyde for 30 min, followed by three washes in PBS. Cells were incubated with the primary rabbit TJP1 (Abcam, ab216880, 1:200, Cambridge, MA, USA), OCLN (Proteintech, 27260-1-AP, 1:200, Chicago, IL, USA), CLDN5 (Abcam, ab15106, 1:200, Cambridge, MA, USA) antibody, or SARS-COV-2 spike antibody (Abclonal, A20022, 1:100, Wuhan, China) overnight at 4 °C, and then incubated with Alexa Fluor 594 goat anti-rabbit antibody (Bioss, bs-0296R-AF594, 1:200, Woburn, MA, USA) for another hour. Cells were counterstained with DAPI (US Everbright Inc., D4080, Suzhou, China) to visualize the nucleus morphology and mounted and photographed using BX41 fluorescence microscopy (Olympus, Tokyo, Japan).

Mouse brain sections were incubated with primary TJP1 (Abcam, ab216880, 1:200, Cambridge, MA, USA), OCLN (Proteintech, 27260-1-AP, 1:200, Chicago, IL, USA), CLDN5 (Abcam, ab15106, 1:200, Cambridge, MA, USA), ICAM1 (Proteintech, 16174-1-AP, 1:200, Chicago, IL, USA), VCAM1 antibody (Abcam, ab134047, 1:200, Cambridge, MA, USA), CD44 antibody (Proteintech, 15675-1-AP, 1:200, Chicago, IL, USA), or SARS-COV-2 spike antibody (Abclonal, A20022, 1:100, Wuhan, China), followed by incubation with secondary antibody conjugated with Cy3 (Beyotime Biotechnology, China). The same sections were then incubated with CD31 (HuaAn Biotechnology, ER31219, 1:200, Hangzhou, China) primary antibody, followed by incubation with the appropriate secondary antibody fluorescein isothiocyanate (Beyotime Biotechnology, China) prior to final nuclear staining with DAPI. The sections were photographed and analyzed using a BX41 microscope (Olympus, Tokyo, Japan).

### Statistical analysis

All data conformed to the Gaussian distribution. Data are expressed as mean ± standard deviation (SD), and the significance of differences between groups was evaluated using multiple *t*-tests. A level of *p* < 0.05 (*) was considered significant, and *p* < 0.01 (**) or *p* < 0.001 (***) were considered extremely significant. Graphs were plotted and analyzed using the GraphPad Prism ver. 6.0 (Graph-Pad Software, La Jolla, CA, USA).

## Results

### SARS-CoV-2 infects brain microvascular endothelial cells

**Fig. 1:**
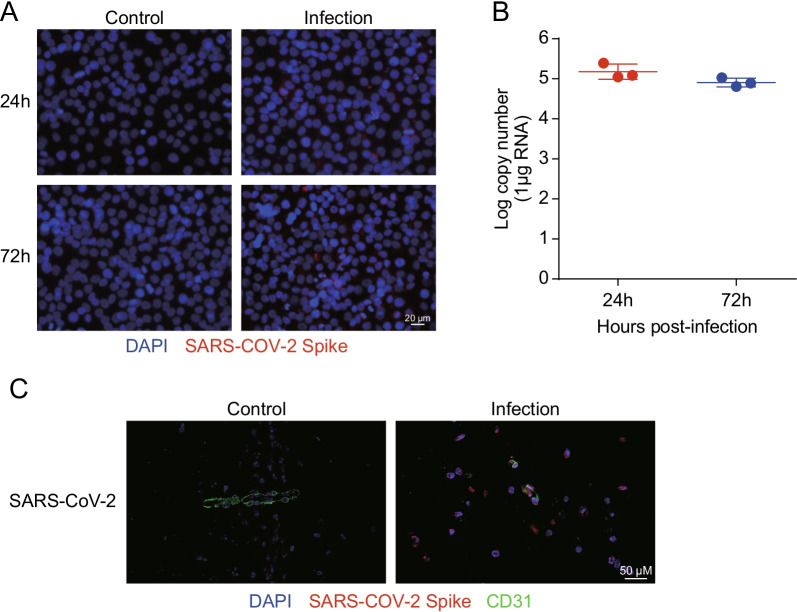
Infection efficiency of SARS-CoV-2 analysis by RT-qPCR and immunofluorescence. **A** Infection efficiency of SARS-CoV-2 was detected using SARS-COV-2 Spike rabbit antibody, by immunofluorescence. Nuclei were stained in blue with DAPI, while SARS-CoV-2 was stained in red. Scale bar, 20 μm. **B** RT-qPCR for the RNA-dependent RNA polymerase (RdRP)-coding gene of SARS-CoV-2. Data are expressed as mean ± SD from three independent experiments. **C** Brain samples of both mice infected with SARS-CoV-2 for 5 d and those without were analyzed to determine the SARS-CoV-2 location by immunofluorescence. Nuclei were stained in blue with DAPI, while SARS-CoV-2 was stained in red. CD31 was specifically applied for labeling the micro-vessels in green. Scale bar indicates 50 μm

RT-qPCR and immunofluorescence were performed to determine the level of SARS-CoV-2 replication in human brain microvascular endothelial cells (hBMECs) at 24 h and 72 h. The hBMECs were infected with SARS-CoV-2 at a MOI of 1 and processed for the examination. As shown in Fig. 1A, SARS-CoV-2 was observed in hBMECs, as demonstrated by SARS-COV-2 spike glycoprotein-specific staining at 24 h and 72 h after infection. To confirm this observation, viral RNA was detected using RT-qPCR (Fig. 1B), and results showed high levels of viral RNA at 24 h and 72 h post-infection, indicating the successful infection of SARS-CoV-2 in hBMECs. In addition, immunofluorescence revealed a SARS-CoV-2 spike glycoprotein-specific fluorescence in SARS-CoV-2-challenged mouse brain. SARS-CoV-2 was stained in red, while CD31 was specifically applied for labeling the micro-vessels in green (Fig. 1C). Together, these results indicate that SARS-CoV-2 possesses the ability to infect hBMECs.

### Identification of differentially expressed mRNAs in hBMECs upon SARS-CoV-2 infection

**Fig. 2:**
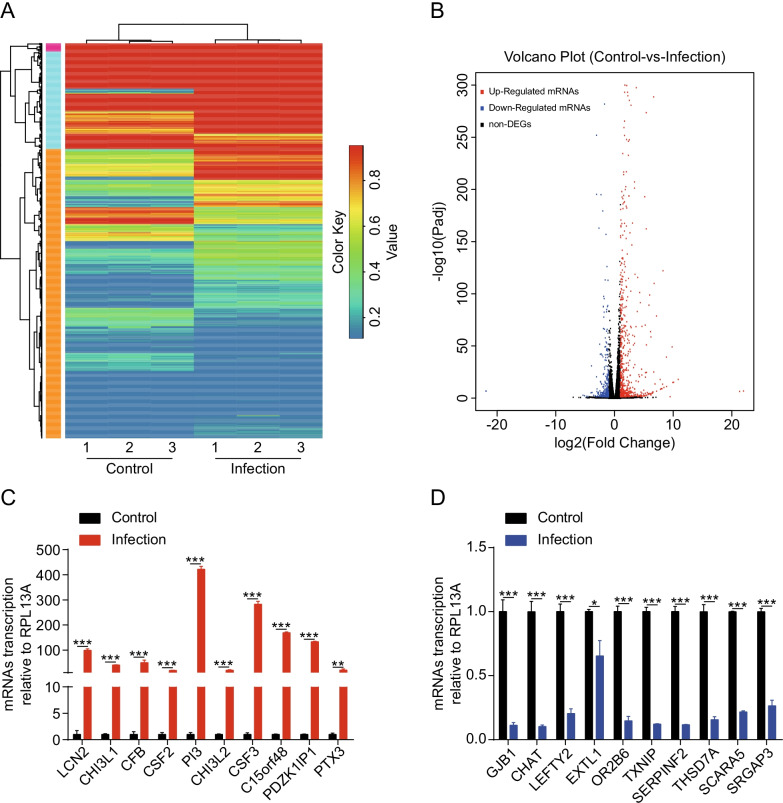
Expression profiling of the changes in messenger RNAs (mRNAs) in SARS-CoV-2-infected human brain microvascular endothelial cells (hBMECs). **A** Heat map showing unsupervised clustering of significantly expressed mRNAs from hBMECs in the SARS-CoV-2 infection groups compared with the control groups. The expression profiles are displayed with three samples in each group. Red represents high, while blue represents low expression, relative to that of the control. **B** Volcano plot of the upregulated and downregulated mRNAs from hBMECs in the SARS-CoV-2 infection group compared with the control group. Increase by ≥ twofold or decrease to ≤ 0.5-fold, *p* < 0.05 was considered statistically significant. **C**, **D** The relative expression levels of 20 mRNAs, including 10 downregulated and 10 upregulated ones, were examined by RT-qPCR in hBMECs infected with SARS-CoV-2 for 72 h and those without. The data from RNA-sequencing (RNA-seq) were highly consistent with the RT-qPCR results. RPL13A was used as the internal reference. Data were presented as the mean ± SD from three independent experiments. *p < 0.05, **p < 0.01, ***p < 0.001

To better understand the mechanism of SARS-CoV-2 infection in hBMECs, we conducted a comparative transcriptomic analysis between the uninfected and infected primary cells. The heat map and the volcano plot revealed the alteration trends of these mRNAs in the cells upon infection with SARS-CoV-2 (Fig. 2A, B). In total, 18,947 mRNAs were identified. Of these, 610 were significantly upregulated and 307 were downregulated (increased by ≥ twofold or decreased by ≤ 0.5-fold, at *p* ≤ 0.05) in SARS-CoV-2-infected hBMECs, compared with non-infected cells (Additional file 2: Table S2). The most significantly upregulated and downregulated mRNAs are listed in Tables 1 and 2, respectively. To verify the results of the differentially altered mRNAs, RT-qPCR was performed on 10 significantly upregulated and 10 significantly downregulated mRNAs that were randomly selected from the most significant changed mRNAs in the sequencing data. The results demonstrated that the upregulation (Fig. 2C) or downregulation (Fig. 2D) trends of these mRNAs were consistent with those from the sequencing data.

**Table 1:** The most significantly upregulated mRNAs in hBMECs upon infection

Gene symbol	Ensembl gene ID	log2(fold change)	*p* value	*q* value
PRRC2A	ENSG00000204469	21.97584681	1.86E−08	1.53E−07
PTDSS2	ENSG00000174915	21.30706691	5.01E−08	3.88E−07
SAA2	ENSG00000134339	10.07835285	3.79E−17	6.57E−16
SAA1	ENSG00000173432	9.932668453	5.99E−17	1.02E−15
LCN2	ENSG00000148346	9.826441157	1.49E−51	8.29E−50
SULT1A4	ENSG00000213648	9.478170754	0.015280772	0.042089886
SFT2D3	ENSG00000173349	8.882246082	5.53E−13	7.05E−12
CHI3L1	ENSG00000133048	8.853778776	1.20E−41	5.21E−40
CFB	ENSG00000243649	8.432931778	6.22E−12	7.35E−11
CCL20	ENSG00000115009	8.275035624	4.64E−125	7.85E−123
CSF2	ENSG00000164400	8.201980672	3.01E−15	4.57E−14
PI3	ENSG00000124102	8.089107263	8.07E−11	8.64E−10
TNF	ENSG00000232810	8.020602424	1.11E−10	1.17E−09
CHI3L2	ENSG00000064886	7.836029091	3.94E−10	3.88E−09
CSF3	ENSG00000108342	7.776493262	7.93E−20	1.59E−18
C15orf48	ENSG00000166920	7.250651063	0	0
SAA2-SAA4	ENSG00000255071	7.18957744	3.05E−08	2.44E−07
PDZK1IP1	ENSG00000162366	7.168359056	2.26E−81	2.17E−79
PTX3	ENSG00000163661	6.916996585	1.80E−15	2.77E−14
CXCL2	ENSG00000081041	6.754935681	8.15E−113	1.19E−110
IL1B	ENSG00000125538	6.695314953	3.74E−27	1.08E−25
CXCL8	ENSG00000169429	6.669732672	3.53E−292	1.71E−289
LTB	ENSG00000227507	6.542726279	2.40E−07	1.70E−06
IL3RA	ENSG00000185291	6.474107802	1.86E−06	1.14E−05
CA9	ENSG00000107159	6.440743779	2.39E−06	1.45E−05
CXCL1	ENSG00000163739	6.365756137	0	0
SMG1	ENSG00000157106	6.227596608	9.18E−06	5.04E−05
ATF6B	ENSG00000213676	6.220025614	0.000252727	0.0010654
CCL2	ENSG00000108691	6.206816673	2.96E−61	2.04E−59
G0S2	ENSG00000123689	6.171508146	1.04E−05	5.69E−05

**Table 2:** The most significantly downregulated mRNAs in hBMECs upon infection

Gene symbol	Ensembl gene ID	log2(fold change)	*p* value	*q* value
NEU1	ENSG00000204386	− 21.88265783	2.17E−08	1.76E−07
CHMP3	ENSG00000115561	− 5.362242755	0.000493786	0.001962402
GJB1	ENSG00000169562	− 5.101785064	0.001368014	0.004937517
CFAP47	ENSG00000165164	− 4.724579521	0.005709798	0.017747683
MGARP	ENSG00000137463	− 4.503180663	0.010783266	0.031122636
CCDC187	ENSG00000260220	− 3.996799492	1.20E−06	7.63E−06
CHAT	ENSG00000070748	− 3.913490411	3.62E−33	1.30E−31
LEFTY2	ENSG00000143768	− 3.808163847	6.57E−07	4.35E−06
novel.1068	–	− 3.551248115	0.000551614	0.002160943
EXTL1	ENSG00000158008	− 3.265373003	0.001404158	0.005049692
OR2B6	ENSG00000124657	− 3.085358052	0.012758699	0.035990702
TXNIP	ENSG00000265972	− 3.066511011	2.61E−255	1.08E−252
SERPINF2	ENSG00000167711	− 3.043253694	1.57E−198	4.88E−196
SLC2A2	ENSG00000163581	− 3.019859147	0.012528758	0.035401966
THSD7A	ENSG00000005108	− 2.990344181	6.00E−08	4.59E−07
OR1F12	ENSG00000220721	− 2.971771113	1.57E−10	1.63E−09
H2AC7	ENSG00000196866	− 2.87703511	0.003118136	0.010338306
LCN12	ENSG00000184925	− 2.834977689	1.15E−06	7.32E−06
novel.19299	–	− 2.832122052	9.66E−06	5.29E−05
H3C4	ENSG00000197409	− 2.772546205	1.60E−06	1.00E−05
CAPN3	ENSG00000092529	− 2.712469596	3.46E−05	0.000173351
H2BC8	ENSG00000273802	− 2.702182842	3.80E−07	2.62E−06
SCARA5	ENSG00000168079	− 2.635582038	5.34E−166	1.15E−163
FOXJ1	ENSG00000129654	− 2.605063889	0.01084363	0.031254005
SMTNL2	ENSG00000188176	− 2.554792408	0.000873797	0.003281935
CDH12	ENSG00000154162	− 2.525048622	3.71E−54	2.22E−52
SRGAP3	ENSG00000196220	− 2.482822207	1.21E−08	1.01E−07
ERP27	ENSG00000139055	− 2.475509651	0.006759849	0.020625759
DNAH6	ENSG00000115423	− 2.439137171	0.008656114	0.025631459
novel.774	–	− 2.410152295	2.27E−44	1.08E−42

### Bioinformatic analysis of the altered mRNAs

**Fig. 3:**
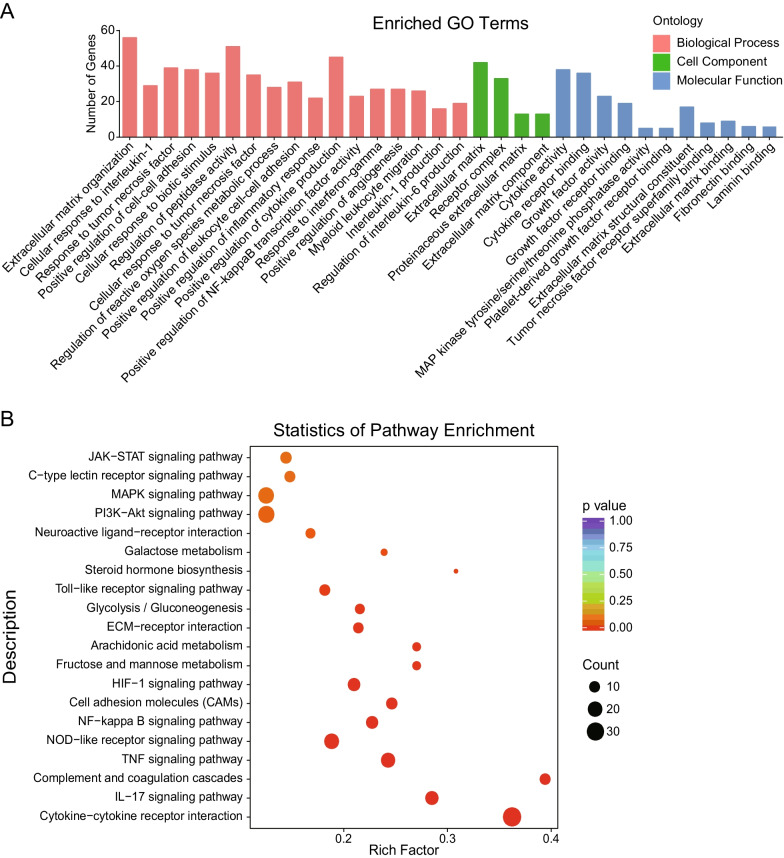
Summary of the Gene Ontology (GO) and Kyoto Encyclopedia of Genes and Genomes (KEGG) pathway analyses for differentially expressed mRNAs. **A** Go of the differentially expressed mRNAs. The *x*-axis represents the enrichment pathway name. The *y*-axis is the targeted gene numbers corresponding to the GO terms. **B** KEGG analysis of the 20 most enriched pathways. The coloring of the *p* values indicates the significance of the rich factor. Circles indicate the target genes that are involved, with sizes proportional to the number of genes. The *x*-axis represents the rich factor. The *y*-axis represents the enrichment pathway name

We next analyzed the potential functions of the 917 differentially expressed mRNAs in SARS-CoV-2-infected hBMECs. These mRNAs were assigned to three GO categories, including biological process, cellular component, and molecular function. The mRNAs that belonged to biological process class were mainly involved in inflammatory reaction (e.g., inflammatory response, cytokine production, NF-κB transcription factor activity, and response to interferon-gamma), leukocyte migration (e.g., positive regulation of leukocyte cell–cell adhesion and myeloid leukocyte migration), and angiogenesis. Those classified as cellular components were mainly divided into extracellular matrix, receptor complex, proteinaceous extracellular matrix, and extracellular matrix component. Lastly, those within the molecular function category were mainly associated with cytokine activity, cytokine receptor binding, growth factor activity, growth factor receptor binding, MAP kinase tyrosine/serine/threonine phosphatase activity, platelet-derived growth factor receptor binding, extracellular matrix structural constituent, tumor necrosis factor receptor superfamily binding, fibronectin binding, and laminin binding (Fig. 3A). The above results implied that the hBMECs were activated and injured in response to SARS-CoV-2.

The signaling pathways enriched by these 917 differentially expressed mRNAs were also determined using Kyoto Encyclopedia of Genes and Genomes (KEGG) analysis, and the results revealed several canonical signaling pathways that were significantly enriched, some of which have already been associated with inflammatory response (e.g., cytokine–cytokine receptor interaction, NOD-like receptor signaling pathway, Toll-like receptor signaling pathway, NF-kappa B signaling pathway, and MAPK signaling pathway), the BBB permeability (e.g., ECM–receptor interaction, IL-17 signaling pathway, TNF signaling pathway, and HIF-signaling pathways), and leukocyte migration (e.g., cell adhesion molecules and PI3K–Akt signaling pathway) (Fig. 3B). Taken together, these results revealed the potential signaling pathways involved in SARS-CoV-2 infections, most of which are involved in the regulation of BBB permeability as well as CNS inflammatory responses.

### SARS-CoV-2 infection induced high levels of inflammatory response in hBMECs

**Fig. 4:**
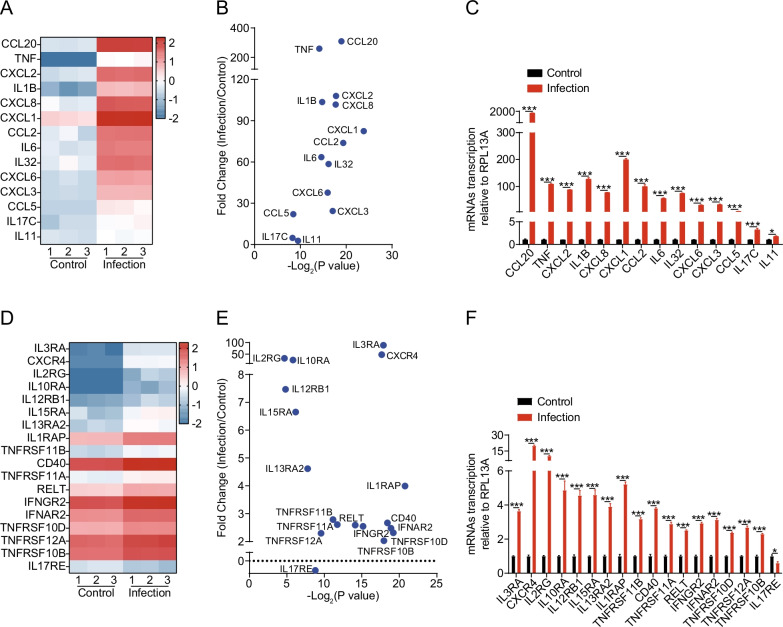
SARS-CoV-2 infection of hBMECs induced a severe inflammatory response. **A** Heat maps depicting virally regulated cytokines and chemokines upon SARS-CoV-2 infection in hBMECs. Colored bar represents expressive abundance of log2 transformed values. **B** Significance analysis and difference multiples of cytokines and chemokines in hBMECs upon SARS-CoV-2 infection. **C** RT-qPCR analysis of cytokines and chemokines transcription in hBMECs upon SARS-CoV-2 infection. RPL13A was used as the internal reference. Data were presented as the mean ± SD from three independent experiments. *p < 0.05, ***p < 0.001. **D** Heat maps depicting virally regulated cytokine receptors upon SARS-CoV-2 infection in hBMECs. Colored bar represents expressive abundance of log2-transformed values. **E** Significance analysis and difference multiples of cytokine receptors in hBMECs upon SARS-CoV-2 infection. **F** RT-qPCR analysis of cytokine receptors transcription in hBMECs upon SARS-CoV-2 infection. RPL13A was used as the internal reference. Data were presented as the mean ± SD from three independent experiments. *p < 0.05, ***p < 0.001

The infection-induced elevation of cytokines and chemokines have also been recognized as an important contributor to CNS damage in various models of neuroinflammation [27, 28]. As shown in Fig. 4A, B, multiple cytokines (e.g., TNF, IL1B, IL6, IL32, etc.) and chemokines (e.g., CCL20, CXCL2, CXCL8, CXCL1, CCL2, etc.) were significantly upregulated in SARS-CoV-2 infected cells. The RT-qPCR was also applied to verify these observations, and results were consistent with the sequencing data (Fig. 4C), further supporting that SARS-CoV-2 infection of hBMECs led to a high level of pro-inflammatory responses.

Since KEGG analysis revealed that the TNF signaling pathway, IL-17 signaling pathway, and cytokine–cytokine receptor interaction were enriched, we subsequently analyzed the expression of these cytokine receptors. As shown in Fig. 4D, E, a total of 17 cytokine receptors were significantly altered in hBMECs upon SARS-CoV-2 infection. Except for IL17RE, the transcription levels of all other cytokine receptors were significantly increased in response to infection (Fig. 4F).

**Fig. 5:**
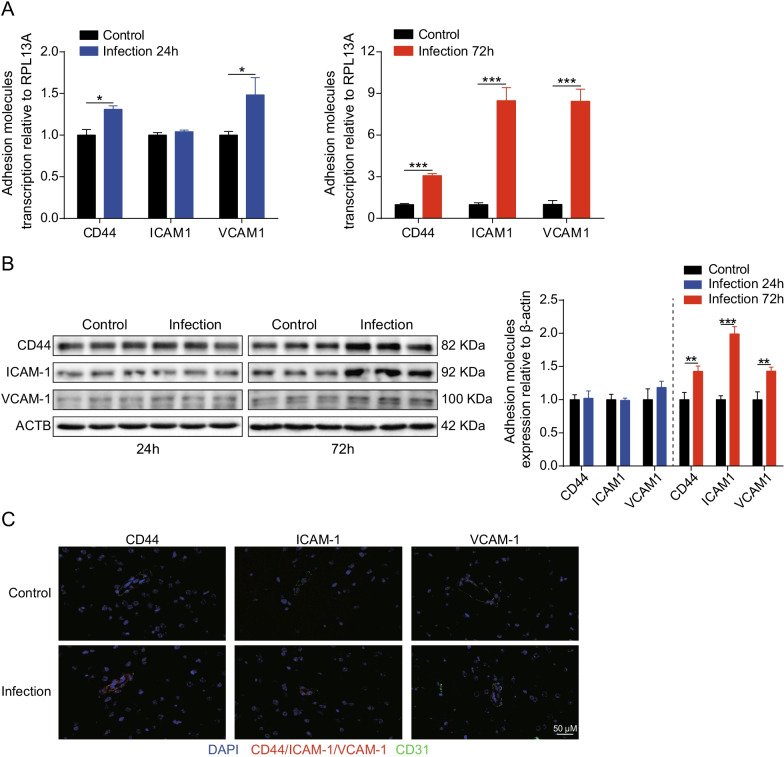
SARS-CoV-2 infection increases expression of adhesion molecules in vitro and in vivo. **A** RT-qPCR analysis of CD44, ICAM1, and VCAM1 transcription in hBMECs 24 and 72 h post-SARS-CoV-2 infection. RPL13A was used as the internal reference. Data were presented as the mean ± SD from three independent experiments. *p < 0.05, ***p < 0.001. **B** Western blot analysis of CD44, ICAM1, and VCAM1 in hBMECs in response to SARS-CoV-2 after 24 and 72 h. β-Actin was used as the loading control, and differences were analyzed by densitometry. **p < 0.01, ***p < 0.001. **C** Brain samples of both mice infected with SARS-CoV-2 for 5 d and those without were analyzed for the expression of adhesion molecules by immunofluorescence. The CD44, ICAM1, and VCAM1 were selected as the markers reflecting the expression of adhesion molecules in red. CD31 was specifically applied for labeling the micro-vessels in green. The cell nucleus was stained in blue with DAPI. Scale bar indicates 50 μm

Moreover, RNA-sequencing data also showed that adhesion molecules, including CD44, ICAM1, and VCAM1, were significantly induced in response to SARS-CoV-2 infection (Additional file 2: Table S2), and we further verified this upregulation in vitro and in vivo. In the hBMECs model, we found that mRNA transcription levels of CD44, ICAM1, and VCAM1 significantly increased at 24 h or 72 h after SARS-CoV-2 infection (Fig. 5A). Similar results were also observed from Western blotting, wherein the expression levels of adhesion molecules in challenged hBMECs were significantly increased after SARS-CoV-2 infection at 72 h, compared to that in control cells (Fig. 5B). In addition, immunofluorescence analysis was performed to further observe the expression of CD44, ICAM1, and VCAM1 in the brains of challenged mice. In infected mice, CD44, ICAM1, and VCAM1 expression was obviously enhanced and distributed around the blood vessels that being labeled with CD31, compared with the control group (Fig. 5C). Taken together, these results indicate that SARS-CoV-2 infection could induce high level of pro-inflammatory responses in hBMECs.

### *SARS-CoV-2 infection damaged the BBB integrity *viadecreasing TJ proteins

**Fig. 6:**
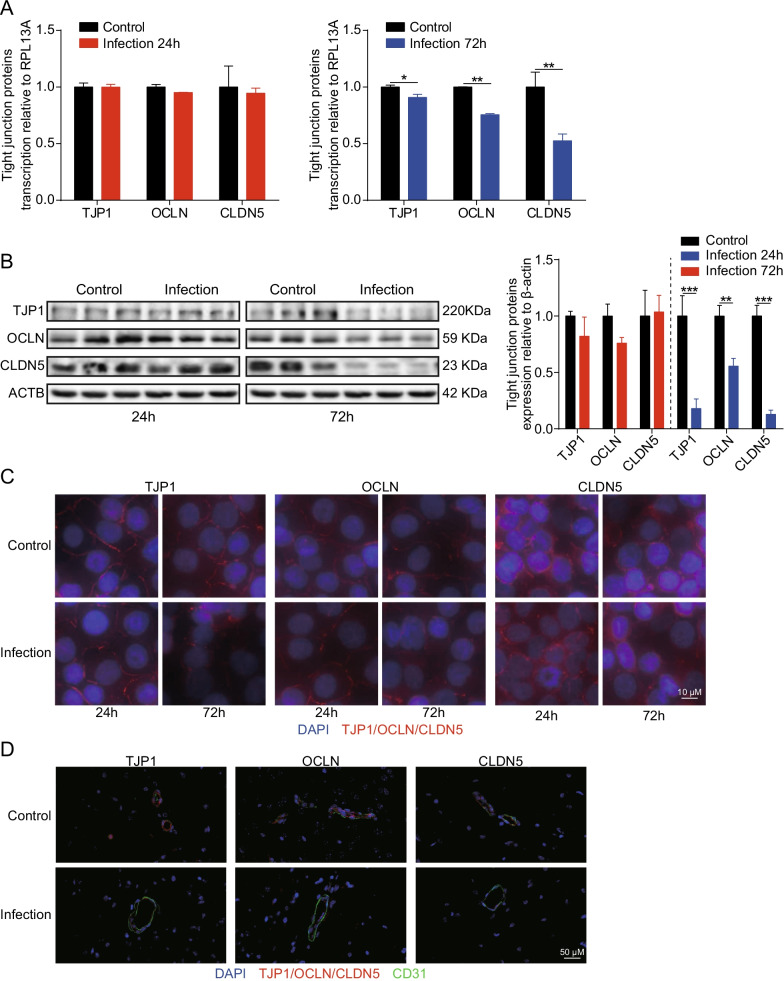
SARS-CoV-2 damaged the integrity of BBB by downregulating and disorganizing TJ proteins. **A** RT-qPCR analysis of TJP1, OCLN, and CLDN5 transcription in hBMECs 24 and 72 h post-infection with SARS-CoV-2. RPL13A was used as the internal reference. Data were presented as the mean ± SD from three independent experiments. *p < 0.05, **p < 0.01. **B** Western blot analysis of TJP1, OCLN, and CLDN5 in hBMECs in response to SARS-CoV-2 at 24 and 72 h post-infection. β-Actin was used as the loading control, and differences were analyzed by densitometry. **p < 0.01, ***p < 0.001. **C** Immunofluorescence analyses of TJP1, OCLN, and CLDN5 expression and distribution in hBMECs 24 and 72 h after infection with SARS-CoV-2. Nuclei were stained in blue with DAPI, while TJP1, OCLN, and CLDN5 were stained in red. Scale bar, 10 μm. **D** Brain samples of both mice infected with SARS-CoV-2 for 5 d and those without were analyzed for the integrity of vascular endothelium by immunofluorescence. TJ proteins, TJP1, OCLN, and CLDN5 were selected as the markers reflecting the integrity of the vascular endothelium in red. CD31 was specifically applied for labeling the micro-vessels in green. The cell nucleus was stained in blue with DAPI. Scale bar indicates 50 μm

We next wondered whether SARS-CoV-2 infection could disrupt the BBB. Since TJ proteins are recognized as key components of monolayer hBMECs and determine the permeability of the BBB, we monitored the alteration of these TJ proteins (e.g., TJP1, OCLN, and CLDN5) in monolayers hBMECs at 24 h and 72 h post-SARS-CoV-2 infection, by RT-qPCR and Western blotting. We found the mRNA transcription levels of TJP1, OCLN, and CLDN5 were unaffected by the infection at 24 h, but were significantly decreased after 72 h of SARS-CoV-2 infection (Fig. 6A). Similar results were also obtained from Western blotting, wherein the expression levels of TJ proteins at 72 h were significantly lower in hBMECs infected with SARS-CoV-2 than in control cells (Fig. 6B). Immunofluorescence also supported that these TJ proteins were well arranged and distributed around the uninfected hBMECs, while in contrast they were inconsecutive, irregularly distributed, or scattered around the cells 72 h after SARS-CoV-2 infection, indicating the breakdown of TJ proteins between the adjacent endothelial cells (Fig. 6C). In vivo, the distribution of these TJ proteins in the brain of mice was additionally examined, as seen in Fig. 6D. The in situ immunofluorescence revealed that the TJ proteins (TJP1, OCLN, and CLDN5) were well-expressed and distributed around the brain vessels of the control mice, whereas they were inconsecutively distributed, irregular, or gapped in vascular endothelial layer in the brains of challenged mice, further evidencing the disruption of TJ proteins between adjacent endothelial cells. Together, these findings firmly suggest that SARS-CoV-2 infection induced BBB disruption by downregulating the expression of TJ proteins as well as by altering their distribution.

## Discussion

With mounting infections, fatalities, and economic losses caused by SARS-CoV-2, understanding the pathogenesis of SARS-CoV-2 is imperative. Aside from the lungs, other tissues and organs, such as the heart, liver, kidneys, spleen, hilar lymph nodes, bone marrow, and even brain tissues are also affected in patients with COVID-19 [29–31]. Notably, neurological and psychiatric symptoms have been reported in patients with SARS-CoV-2 infection [32, 33]. A case of CNS involvement in a patient infected with SARS-CoV-2, exhibiting meningitis and encephalitis, was confirmed by SARS-CoV-2 RT-qPCR by using the patient’s cerebrospinal fluid [34]. Recent studies have also demonstrated the ability of SARS-CoV-2 to infect CNS cells, especially the BMECs of the BBB [35]. Experiments also reveal that SARS-CoV-2 spike protein altered human BBB function by inducing degradation of endothelial TJ proteins [13, 14]. Therefore, this study aimed to explore the essential host mRNAs involved in SARS-CoV-2 infection of hBMECs.

**Fig. 7:**
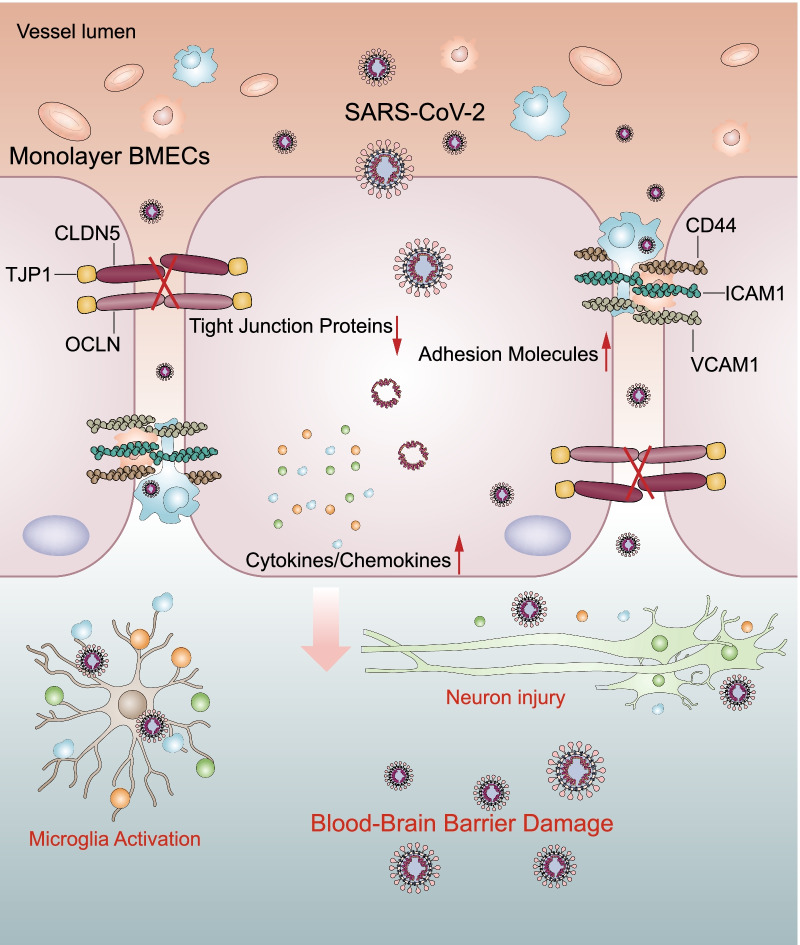
Schematic representation of SARS-CoV-2-induced BBB damage and CNS dysfunctions. SARS-CoV-2 infection induced production of multiple cytokines, chemokines, and adhesion molecules in hBMECs. Meanwhile, SARS-CoV-2 infection could increase the BBB permeability, by downregulating as well as remodeling the intercellular TJ proteins

Using the RNA-seq approach, the differentially expressed mRNAs in hBMECs in response to SARS-CoV-2 were identified. Expression levels of 927 mRNAs were significantly changed in response to infection; among which, 610 were significantly increased while 317 mRNAs were decreased. In addition, GO and KEGG enrichment analysis of these differentially expressed mRNAs illustrated the potential roles of the altered mRNAs in SARS-CoV-2 pathogenesis in hBMECs. Moreover, in vivo and in vitro experiments demonstrated that SARS-CoV-2 damaged the integrity of the BBB by negatively regulating the expression of TJ proteins, such as TJP1, OCDN, and CLDN5. Meanwhile, the SARS-CoV-2 induction of cytokines, chemokines, and adhesion molecules largely promoted the development of the CNS inflammation response (Fig. 7). To the best of our knowledge, this is the first demonstration of the differential induction of host mRNAs by SARS-CoV-2 in hBMECs, providing a theoretical basis for future studies on BBB damage caused by SARS-CoV-2.

Several studies have reported that the excessive inflammatory response induced by SARS-CoV-2 infection is a major cause of disease severity and death in infected patients [36–38]. Moreover, induction of the endothelial adhesion molecules is widely accepted as an important indicator of endothelial cell activation, which causes the recruitment of macrophages and monocytes that are responsive to the infection, and release cytokines and prime adaptive T and B cell immune responses [39]. Similarly, in this study, we found that the expression levels of CD44, ICAM1, and VCAM1 were significantly elevated in SARS-CoV-2-infected hBMECs and in the brains of SARS-CoV-2-infected mice, indicating that there would be an increased recruitment of mononuclear leukocytes from the periphery to the CNS.

Additionally, increased levels of several cytokines and chemokines, including TNF, IFNG, IL1B, IL2, IL6, IL7, IL8, IL9, IL10, IL17A, CSF3, CCL2, IP10, MCP3, and macrophage inflammatory protein 1α have been reported in patients infected with SARS-CoV-2 [40–42]. Consistent with these observations, we also found the increased amounts of chemokines and cytokines, including CCL20, TNF, CXCL2, IL1B, CXCL8, CXCL1, CCL2, IL6, IL32, CXCL6, CXCL3, CCL5, IL17C, and IL11, in hBMECs in response to SARS-CoV-2 infection. This massive production of these pro-inflammatory molecules will trigger the development of the CNS cytokine storm, which is the most important factor in mediating the BBB dysfunction [43]. More importantly, the cytokine storm could further induce the activation of microglia and astrocytes, as well as the neuronal injury, finally leading to neurodegenerative disorders and sequelae [44]. Of note, high levels of IL1B and IL6 have been linked to the worse prognosis in patients with COVID-19 [45, 46].

Based on the KEGG enrichment analysis, we also noticed that the JAK–STAT-, MAPK-, HIF-1-, NF-κB-, NOD-like receptor-, TNF-, and IL-17-signaling pathways, which are commonly recognized as inflammatory amplification loops that cause BBB dysfunction, were activated in SARS-CoV-2-infected hBMECs [47–50]. Meanwhile, genes associated with cytokine receptors, such as IL3RA, CXCR4, CXCL12, IL2RG, IL10RA, IL12RB1, IL15RA, IL13RA2, IL1RAP, TNFRSF11B, CD40, TNFRSF11A, RELT, IFNGR2, IFNAR2, TNFRSF10D, TNFRSF12A, and TNFRSF10B, were all found to be upregulated in the infected cells. Among these, IFNGR2 and IFNAR2 encode components of the IFNG receptor complex. TNFRSF11B, CD40, TNFRSF11A, RELT, TNFRSF10D, TNFRSF12A, and TNFRSF10B are members of the TNF receptor superfamily. Given the increased levels of TNF and IFNG in the plasma of patients with COVID-19, we inferred that TNF- and IFNG signaling pathways were likely to be activated in SARS-CoV-2-infected hBMECs. Therefore, the inhibition of inflammatory signaling in CNS may be a beneficial strategy in treating SARS-CoV-2 infection.

The BBB serves as a physical and physiological barrier against the entry of cells and molecules into CNS [7]. Despite the highly restrictive nature of the CNS, some viruses, such as those causing rabies, Japanese encephalitis, SARS, MERS, and dengue, employed different strategies to break through the BBB, thereby invading the CNS and causing encephalitis [51–54]. BMECs are the most direct and functional structural components of the BBB, and these are tightly interconnected by the formation of TJ proteins, which regulate the permeability of the BBB. In general, there are two possible mechanisms for the spread of neurotropic virus across the BBB. The first mechanism involves the infection of and the subsequent transport of viral genetic material across vascular endothelial cells [55], and the second one is termed as the “Trojan horse mechanism” which involves the infection of leukocytes that help to pass through the BBB [56]. Once the virus enters host CNS, it continues the life cycle of viral budding, allowing further infection of neurons, glia, and microglia [57]. In the present study, our data supported that hBMECs allowed SARS-CoV-2 infection, but no evidence supporting viral replication was observed. However, we demonstrated that SARS-CoV-2 invasion of the brain increased the BBB permeability by downregulating and redistributing TJ proteins. Therefore, damaging the BBB integrity could be an important strategy employed by SARS-CoV-2 in order to cause host CNS infection.

Several host molecules have been reported to mediate BBB dysfunction by regulating TJ proteins in CNS infection, including ROS generation, upregulation of matrix metallopeptidases (MMPs; e.g., MMP3, MMP7, MMP8, and MMP9), cytokines (e.g., IL1B, IL6, IL17, and TNF), chemokines (e.g., CXCL1 and CXCL10), growth factors (e.g., VEGF, PDGF, and ANGPTL4), transcription factors (e.g., SNAI1 and EGR1), and activation of Rho kinase [27, 58–62]. In the current study, we demonstrated that SARS-CoV-2 infection induced high levels of inflammatory responses in hBMECs, suggesting that the induction of severe CNS inflammation and systemic inflammatory responses may be an important contributor influencing the BBB integrity in SARS-CoV-2 infection. More importantly, we also found the significant induction of MMP3, MMP7, MMP9, VEGFA, PDGFA, PDGFB, ANGPTL4, and SNAI1 in the challenged hBMECs (Additional file 3: Table S3). This prompted us to believe that these pro-inflammatory factors, MMP family, and growth factors, derived from the brain or the periphery, could promote disruption of the BBB in response to SARS-CoV-2 infection. Interestingly, a recent study found that SARS-CoV-2 RNA was observed in the BMECs, perivascular space and vascular wall in the infected K18-hACE2 transgenic mice. And the damage of BBB integrity was also found in the infected mice. But the expression and the ultrastructure of TJP1, OCLN, and CLDN5 were shown to be unchanged, whereas, the basement membrane was disrupted [63]. Therefore, the molecular mechanisms of the damage of BBB integrity caused by SARS-CoV-2 infection still need further study. However, the CNS cytokine storm induced by SARS-CoV-2 should be one of the key factors to help it destroy the integrity of the host BBB.

## Conclusions

In summary, we showed here that BMECs, which comprise the most important component of BBB, were susceptible to SARS-CoV-2 infection. Our findings highlighted that SARS-CoV-2 infection could lead to BBB disruption as well as the CNS inflammatory responses, which are the two essential aspects determining the occurrence and development of CNS infection and outcomes. Moreover, as SARS-CoV-2 infection is productive in endothelial cells, which can allow the entry of the virus into CNS and facilitate the infection of microglia and neurons within CNS parenchyma. Understanding the mechanisms of SARS-CoV-2-induced BBB dysfunction may provide new concepts for further therapeutic intervention in COVID-19.

## Supplementary Information

**Additional file 1: Table S1.** Primers used for qPCR in this study.

**Additional file 2: Table S2.** Differentially expressed mRNAs (Control vs. Infection).

**Additional file 3: Table S3.** Tight junction proteins related mRNAs.

## Data Availability

The data that support the findings of this study are available from the corresponding author upon reasonable request.

## Abbreviations

SARS-CoV-2: Severe acute respiratory syndrome coronavirus 2
BBB: Blood–brain barrier
hBMECs: Human brain microvascular endothelial cells
CNS: Central nervous system
COVID-19: Coronavirus disease 19
BMECs: Brain microvascular endothelial cells
TJ: Tight junction
CLDNs: Claudins
OCLN: Occludin
VCAM-1: Vascular endothelial cell adhesion molecule 1
ICAM-1: Intercellular cell adhesion molecule 1
PBS: Phosphate-buffered saline
KEGG: Kyoto Encyclopedia of Genes and Genomes
GO: Gene Ontology
BLAST: Basic local alignment search tool
RT-qPCR: Reverse transcription and real-time quantitative polymerase chain reaction
cDNA: Complementary DNA
SD: Standard deviation
