# Supplementary material for: SARS-CoV-2 productively infects human brain microvascular endothelial cells
Source: J Neuroinflammation. 2022 Jun 15;19:149. doi: 10.1186/s12974-022-02514-x (PMC9198209; doi:10.1186/s12974-022-02514-x)
Supplement: Supplementary file 3 — Additional file 3: Table S3. Tight junction proteins related mRNAs. [file 12974_2022_2514_MOESM3_ESM.docx]

Table S3. Tight junction proteins related mRNAs

| **Gene Symbol** | **Ensembl Gene ID** | **log2(Fold Change)** | **pvalue** | **qvalue** |
| --- | --- | --- | --- | --- |
| MMP7 | ENSG00000137673 | 4.412403272 | 0.003751992 | 0.012181916 |
| MMP3 | ENSG00000149968 | 2.449865048 | 0.018517529 | 0.049732717 |
| MMP9 | ENSG00000100985 | 2.439229619 | 0.001954853 | 0.00679785 |
| ANGPTL4 | ENSG00000167772 | 2.359312342 | 1.8422E-249 | 6.98E-247 |
| VEGFA | ENSG00000112715 | 2.284973376 | 3.2206E-293 | 1.6056E-290 |
| PDGFB | ENSG00000100311 | 1.261567366 | 9.6959E-107 | 1.3507E-104 |
| PDGFA | ENSG00000197461 | 1.236906384 | 2.67469E-14 | 3.76463E-13 |
| SNAI1 | ENSG00000124216 | 1.021568241 | 1.28299E-09 | 1.19795E-08 |
